# COVID-19 in the Clinic: Human Testing of an Aerosol Containment Mask for Endoscopic Clinic Procedures

**DOI:** 10.1177/01945998211029184

**Published:** 2021-07-27

**Authors:** Elisabeth H. Ference, Wihan Kim, John S. Oghalai, Clayton B. Walker, Jee-Hong Kim, Tyler Gallagher, Harrison J. Ma, Brian E. Applegate

**Affiliations:** 1 Caruso Department of Otolaryngology–Head and Neck Surgery, Keck School of Medicine of University of Southern California, Los Angeles, California, USA; 2 Keck School of Medicine of University of Southern California, Los Angeles, California, USA

**Keywords:** negative-pressure mask, endoscopy, laryngoscopy, nasal endoscopy, aerosol production, cough, sneeze, Rainbow Passage, virus, COVID-19

## Abstract

**Objective:**

To create an aerosol containment mask (ACM) for common otolaryngologic endoscopic procedures that also provides nanoparticle-level protection to patients.

**Study Design:**

Prospective feasibility study .

**Setting:**

In-person testing with a novel ACM.

**Methods:**

The mask was designed in Solidworks and 3D printed. Measurements were made on 10 healthy volunteers who wore the ACM while reading the Rainbow Passage repeatedly and performing a forced cough or sneeze at 5-second intervals over 1 minute with an endoscope in place.

**Results:**

There was a large variation in the number of aerosol particles generated among the volunteers. Only the sneeze task showed a significant increase compared with normal breathing in the 0.3-µm particle size when compared with a 1-tailed *t* test (*P* = .013). Both the 0.5-µm and 2.5-µm particle sizes showed significant increases for all tasks, while the 2 largest particle sizes, 5 and 10 µm, showed no significant increase (both *P* < .01). With the suction off, 3 of 30 events (2 sneeze events and 1 cough event) had increases in particle counts, both inside and outside the mask. With the suction on, 2 of 30 events had an increase in particle counts outside the mask without a corresponding increase in particle counts inside the mask. Therefore, these fluctuations in particle counts were determined to be due to random fluctuation in room particle levels.

**Conclusion:**

ACM will accommodate rigid and flexible endoscopes plus instruments and may prevent the leakage of patient-generated aerosols, thus avoiding contamination of the room and protecting health care workers from airborne contagions.

**Level of evidence:**

2

As SARS-COV-2, the virus responsible for COVID-19, continues to spread around the world, there is a need to be able to perform both rigid and flexible endoscopy on patients with active or recent COVID-19 or if the patient’s COVID status is unknown. While the number of new cases has fallen with an increase in the number of vaccines, clinicians still face concerns over transmission due to variants or due to patients, such as those with immunosuppression, who may become symptomatic despite vaccination. Speaking, sneezing, and coughing during laryngoscopy and nasal endoscopy are aerosol-generating events.^1,2^ Aerosolized particles may remain viable in the air for hours, placing at risk not only surgeons and staff but also future patients who enter the clinic room.^3^

Authors of previous studies have suggested the use negative-pressure microenvironments,^4^ modification of AMBU,^5^ nasotracheal intubation^6^ face masks with negative pressure, or modified N95 masks^2^ to decrease aerosol dispersion during diagnostic nasal endoscopy and laryngoscopy. We present a 3D-printed negative-pressure respiratory aerosol containment mask (ACM) that also provides N95-level protection to the patient. The negative pressure is generated using a standard suction commonly found in otolaryngology clinics. We measured aerosol generation while using the ACM in healthy volunteers.

## Materials and Methods

The study was approved by the University of Southern California Institutional Review Board (IRB: HS-20-00482). All patients provided written informed consent.

### Mask Design and Development

The mask was designed by the authors and created using Solidworks (Dassault Systemes, Paris, France) and printed using a 3D printer (Ultimaker, Utrecht, The Netherlands) using tough polylactic acid. Initial prototypes were tested by the authors on endoscopic surgery model heads to gauge access to the nasal cavity and the ability to contain aerosols, and these experiments were reported in a separate article.^7^

**Figure 1.**
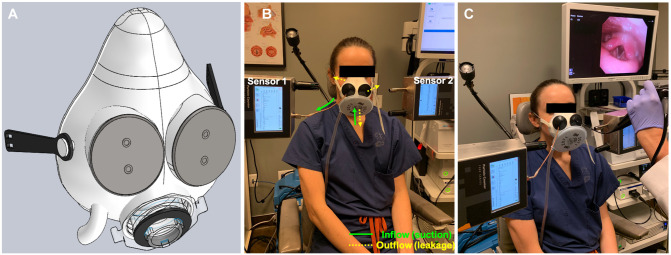
Mask testing setup in a human volunteer. (a) Mask design. (b) Setup for human volunteer. (c) Human volunteer undergoing rigid endoscopy.

The final design included a 3D-printed body with 4 ports, a gel cushion for seal and comfort of fit, and custom blind grommets placed in 2 front ports plus a head strap (**Figure 1**). Each of the blind grommets contains 2 openings, through which an endoscope or suction can be passed. All materials were cleaned in Cidex OPA (Advanced Sterilization Products, Irvine, California). An N95-level commercially available respirator filter can be attached to any of the 3 front ports and replaced between patients. A suction is attached to the suction port of the mask from a commercially available suction pump.

### Testing on Humans

Baseline ambient particle levels were measured with the volunteer wearing the mask, with and without suction. Particle measurements were also obtained with and without a rigid endoscope being placed through the grommets. We obtained particle counts within the mask by threading a 3-mm copper tube through the grommet and attaching it to the input port of the particle counter (sensor 1). Outside the mask, particle counts were obtained by placing the particle counter approximately 2 cm anterior to the grommet where the endoscope was inserted (sensor 2; **Figure 1**). These particle counts were captured at a rate of 0.1 cubic feet every 1 second and sampled at 1 Hz.

Volunteers were asked to read the Rainbow Passage^2^ and the Consensus Auditory-Perceptual Evaluation of Voice^8^ for 1 minute, cough for 1 minute (1 cough every 5 seconds), and simulate a sneeze for 1 minute (1 sneeze every 5 seconds). These tasks were performed with a rigid endoscope placed through a grommet opening to simulate endoscopy. Before and in between tasks, the mask was evacuated using the suction pump, and new baseline measurements were obtained while the volunteer breathed normally. Finally, the volunteer underwent both rigid and flexible endoscopy to ensure that all relevant anatomy could be reached using a 3-pass rigid endoscopy method^9^ and a flexible endoscopy method to visualize the vocal folds and both piriform sinuses. At the conclusion of the trial, the scoping surgeon filled out a Likert-scale survey regarding the ability to see all relevant anatomy, and the volunteer filled out a survey regarding comfort with and without suction.

### Statistical Analysis

Standard *t* tests, as fully specified in the text with α = .05, were used to test for statistical significance. All statistics were calculated using OriginLab (OriginLab Corporation, Northhampton, Massachusetts).

## Results

**Figure 2.**
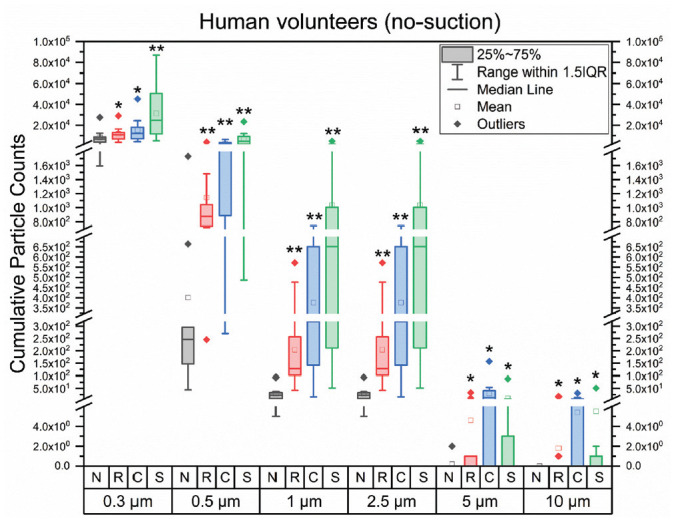
Box plots of cumulative particle counts at sensor 1 measured over 1 minute in human volunteers wearing the aerosol containment mask with no suction. Abbreviations: C, cough; N, normal breathing; R, Rainbow; S, sneeze.

Ten volunteers were recruited for the study. Volunteers presented with a variety of face shapes and sizes, and 3 of the volunteers had facial hair. Each volunteer had a different baseline because of variations in particle counts with normal breathing. Therefore, we made statistical comparisons between the distribution of raw sensor particle counts during normal breathing compared with 3 tasks: reading of the Rainbow Passage, forced cough, and forced sneeze with suction on and off (n = 60). The largest changes occurred in the 0.3-µm particle count, which is why we have focused our analysis on this particle size (**Figure 2**).

We looked at the cumulative particle count measured with sensor 1 (inside mask) during each procedure (**Table 1**). The particle count in sensor 1 measured without suction should be proportional to the total potential aerosol exposure of health care workers in close proximity to the patient. **Figure 2** is a box plot of the total particle count measured over 1 minute for normal breathing and the tasks noted above. For all particle sizes except 5 and 10 µm, the median particle counts increased from left to right, with the lowest particle count being normal breathing and the highest being sneezing. Despite this trend, only the sneeze task showed a significant increase compared with normal breathing in the 0.3-µm particle size when compared with a 1-tailed *t* test (*P* = .013). Both the 0.5-µm and 2.5-µm particle sizes showed significant increases for all tasks (both *P* < .01), whereas while the 2 largest particle sizes, 5 and 10 µm, showed no significant increase. The 1-µm particles showed a significant increase for all tasks except sneeze (P = .03). As noted above, there was large variation in the number of aerosol particles generated among different volunteers, demonstrated by the wide 50th percentile boxes in **Figure 2**. This variation explains why, although we can see peaks corresponding to, for instance, the cough task in the time-domain data of the 0.3-µm particle size for an individual volunteer, the total particle count compared over all volunteers does not rise to statistical significance.

**Table 1.** Statistics for Cumulative Particle Count for the 10 Volunteers.

Size	Task	Mean	SD	SEM	*P* value	Label
0.3 μm	Normal	8074.6	7511.669	2375.398		
	Rainbow	11703.2	7163.463	2265.286	.28	^a^
	Cough	15324.4	12256.27	3875.773	.13	^a^
	Sneeze	31487.8	25959.05	8208.972	.01	^b^
0.5 μm	Normal	400.5	495.7596	156.773		
	Rainbow	1146	962.7876	304.4602	.04	^b^
	Cough	2295.7	1885.602	596.2798	.006	^b^
	Sneeze	6535	7061.98	2233.194	.01	^b^
1 μm	Normal	27.4	25.91953	8.19648		
	Rainbow	203.9	180.5851	57.10603	.006	^b^
	Cough	376	268.8436	85.01582	.001	^b^
	Sneeze	1036.4	1369.32	433.0169	.03	^a^
2.5 μm	Normal	27.4	25.91953	8.19648		
	Rainbow	203.9	180.5851	57.10603	.02	^b^
	Cough	376	268.8436	85.01582	.02	^b^
	Sneeze	1036.4	1369.32	433.0169	.02	^b^
5 μm	Normal	0.2	0.63246	0.2		
	Rainbow	4.6	10.54303	3.334	.20	^a^
	Cough	30	48.35517	15.29125	.07	^a^
	Sneeze	10	27.49949	8.6961	.27	^a^
10 μm	Normal	0	0	0		
	Rainbow	1.8	5.34997	1.69181	.36	^a^
	Cough	5.4	9.97998	3.15595	.12	^a^
	Sneeze	5.5	16.00174	5.06019	.31	^a^

The *P* value and statistical significance were generated by a one-tailed t-test with alpha = 0.05, where we compared the cumulative count for normal breathing to each task.

a No significant difference.

b Significant difference in particle count for a task compared to normal breathing.

**Figure 3.**
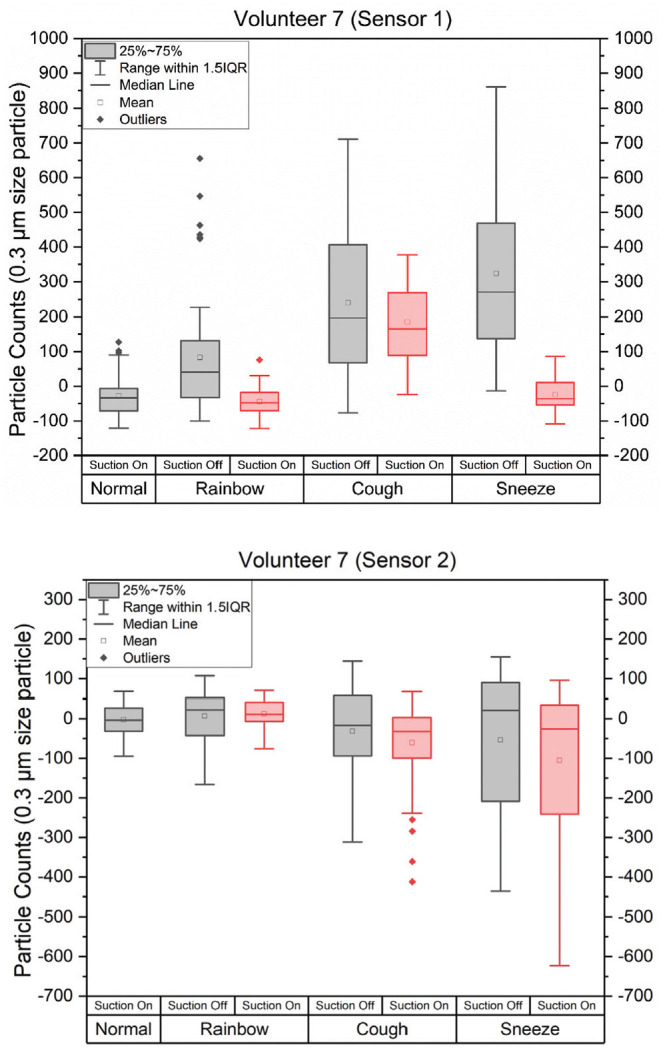
The 0.3-µm particle counts for a representative healthy volunteer for normal breathing, Rainbow Passage, coughing, and sneezing. Inside aerosol containment mask (sensor 1) and outside (sensor 2).

**Figure 4.**
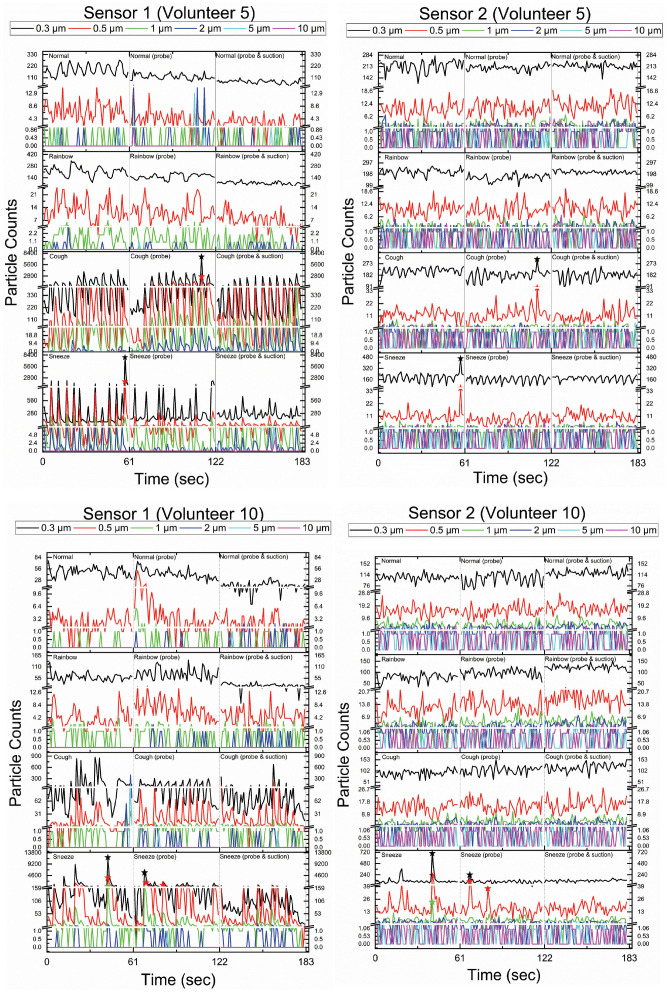
Particle count as a function of time for 2 volunteers. Sensor 1 is inside the mask. Sensor 2 is outside the mask. The leaks are marked by (↔) on both sensor plots.

**Figure 3** is a representative set of box plots for the 0.3-µm particle counts from 1 volunteer (volunteer 7). The statistical data for 0.3-µm particle size on each volunteer is provided in Supplemental Table S1. We used a 1-tailed paired *t* test to compare measurements of normal breathing and each task (Rainbow, cough, sneeze) in sensor 1 and sensor 2 for each volunteer. For all tasks, we measured a statistically significant increase (Supplemental Table S1) in particle count at sensor 1 compared with normal breathing for all volunteers. In 16 of 30 tasks in which the suction was on, there was a significant difference between the particle count measured at sensor 2 during normal breathing and the task. For most of these tasks, particle counts were lower outside the mask compared with normal breathing, similar to what we observed in the mannequin head experiments with suction on. In 2 of the tasks (Rainbow Passage and cough), particle counts were higher outside of the mask, which may indicate a leak. In no cases did the time domain data show peaks in the particle count at sensor 2 that correlated with peaks seen in sensor 1. By this criterion, 0 of the 30 tasks resulted in mask leakage with the suction on. With the suction off, a similar analysis showed 3 cases in which the mask leaked. Both sensor 1 and sensor 2 had increases in particle counts during the same time period. Two volunteers showed leaks during the cough task and 1 during the sneeze task (**Figure 4**).

All volunteers found the mask “very comfortable” with the negative pressure turned on, and 1 of found it “somewhat comfortable,” while the remaining 9 participants found it “very comfortable” without negative pressure (Supplemental Figure S1). The surgeon reported that she was able to visualize all anatomic areas for all volunteers and that the mask was easy to secure for all volunteers.

## Discussion

The ACM prevented the spread of aerosol particles in healthy volunteers. It allowed access to all relevant anatomy using a rigid nasal endoscope and a flexible laryngoscope. In addition, volunteers found the ACM comfortable, especially when the suction was turned on, likely because the addition of the suction overcomes the resistance of breathing through the N95 filter material.

The current study and its design has several limitations. The mask material is not clear. This necessitates scope guidance via a camera or the eye piece to drive the scope from the entrance of the mask into the nares. Future versions of the mask could be made with clear material through injection molding or chemical polishing of transparent 3D-printed parts. As currently designed, the mask could be produced either in the United States or in developing countries with a standard 3D printer and easily obtained disposable parts. All parts of the mask, with the exception of the N95 filter, can be sterilized and reused. The design of the mask is compatible with inexpensive injection molding if mass production is required. Only a single surgeon performed all of the endoscopies; however, an ongoing clinical trial includes surgeons from across all divisions of the otolaryngology department. In addition, the forced sneeze and cough scenario with volunteers may not adequately simulate patients sneezing and coughing during a procedure; however, a larger study on patients in a clinic setting is ongoing. Only 10 volunteers were tested during this study. The study has a 95% power to detect a difference inside the mask between a cough and normal breathing for 0.5-µm particles. However, the 0.3-µm particles had a much higher standard deviation of cumulative levels, and the 5-µm and 10-µm levels had much smaller cumulative particle counts; thus, the study is not adequately powered to determine a difference at the lowest and highest particle sizes. A clinical trial of 100 patients is ongoing.

Because the trials were performed in a regular clinic room, there were significant variations in room particle levels. Prior bench testing of the ACM had been performed in a hood in a laboratory, which could be filtered between trials; however, the hood was too small to contain a human volunteer. We felt that an increase in sensor 2 without a corresponding increase in particles at sensor 1 was likely because of variation in the overall room particle levels associated with the room heating, ventilation, and air conditioning system or with fluctuations in the particle levels in the overall clinic. However, in the 2 of 30 tasks with the suction on associated with an increase in particles at sensor 2 may indicate a slow leak from the mask. We believe this is unlikely, as the 3 of 30 tasks with the suction off where the particle counts were higher at sensor 2 were clearly associated temporally with a rapid increase due to an event (sneeze or cough) captured by sensor 1 (**Figure 4**). In comparison, in volunteer 6 with the suction on, the particle counts decreased inside the mask whereas the particle counts increased outside the mask with reading the Rainbow Passage (Supplemental Table 1). In volunteer 8 with the suction on, the particle counts increased both inside and outside the mask, but there was no temporal relationship to coughs, as seen in **Figure 4**. This evidence, along with the evidence acquired in the validation measurements on mannequins in which no leak was measured when suction was used even when a grommet was removed, lead us to conclude that these events are most likely due to changes in the ambient room particle count rather than a slow leak.^7^ A much larger study currently in progress on patients undergoing endoscopy procedures will further test the ability of the mask to contain aerosols.

We the mask only with healthy volunteers; hence, it is uncertain how patients with otolaryngologic diseases or altered anatomy will tolerate the mask or alter the fit properties of the mask. When surveyed, all volunteers found the mask comfortable with the suction turned on or off. We will address questions of patient comfort and altered anatomy in a larger clinical trial, currently undergoing enrollment. Only 1 surgeon performed all of the endoscopies; however, more surgeons will be included in the clinical trial to solicit a broader range of opinions on the access afforded by the mask.

While the mask allows access to the nose and oral cavity for diagnostic purposes and single-instrument procedures such as suctioning and hand instruments, it does not allow for insertion of larger objects such as nasal packing without removing one of the grommets. Nevertheless, in a trial on a mannequin with the grommet off, we found that there was no significant increase in aerosols external to the mask with the suction on. It may be possible to uncover a grommet to get wider while still providing good protection to the health care worker. The mask has not yet been tested with curved instrumentation or for multistep procedures. In addition, in the study by Workman et al^2^ of an N95 mask with VENT modification, some contamination occurred after N95 respirator removal. We have not yet tested removal procedures; however, we believe most of the aerosols would be evacuated by the suction pump.

## Conclusion

A negative-pressure mask may allow for the passage of both rigid and flexible endoscopes without leakage of particles outside of the mask. This may help prevent contamination of the room and protect health care workers during viral pandemics that involve airborne contagion. A larger clinical study is ongoing.

## Supplemental Material

sj-docx-1-oto-10.117701945998211029184 – Supplemental material for COVID-19 in the Clinic: Human Testing of an Aerosol Containment Mask for Endoscopic Clinic Procedures Click here for additional data file. Supplemental material, sj-docx-1-oto-10.117701945998211029184 for COVID-19 in the Clinic: Human Testing of an Aerosol Containment Mask for Endoscopic Clinic Procedures by Elisabeth H. Ference, Wihan Kim, John S. Oghalai, Clayton B. Walker, Jee-Hong Kim, Tyler Gallagher, Harrison J. Ma and Brian E. Applegate in Otolaryngology–Head and Neck Surgery

sj-pdf-2-oto-10.117701945998211029184 – Supplemental material for COVID-19 in the Clinic: Human Testing of an Aerosol Containment Mask for Endoscopic Clinic Procedures Click here for additional data file. Supplemental material, sj-pdf-2-oto-10.117701945998211029184 for COVID-19 in the Clinic: Human Testing of an Aerosol Containment Mask for Endoscopic Clinic Procedures by Elisabeth H. Ference, Wihan Kim, John S. Oghalai, Clayton B. Walker, Jee-Hong Kim, Tyler Gallagher, Harrison J. Ma and Brian E. Applegate in Otolaryngology–Head and Neck Surgery

## References

[1] Rameau A Lee M Enver N Sulica L. Is office laryngoscopy an aerosol-generating procedure? Laryngoscope. 2020;130(11):2637-2642. doi:10.1002/lary.28973 32671840 PMC7404375

[2] Workman AD Jafari A Welling DB , et al. Airborne aerosol generation during endonasal procedures in the era of COVID-19: risks and recommendations. Otolaryngol Neck Surg. 2020;163(3):465-470. doi:10.1177/0194599820931805 PMC7251624 32452739

[3] van Doremalen N Bushmaker T Morris DH , et al. Aerosol and surface stability of SARS-CoV-2 as compared with SARS-CoV-1. N Engl J Med. 2020;382(16):1564-1567. doi:10.1056/nejmc2004973 32182409 PMC7121658

[4] Hoffman HT Miller RM Walsh JE Stegall HR Diekema DJ. Negative pressure face shield for flexible laryngoscopy in the COVID-19 era. Laryngoscope Investig Otolaryngol. 2020;5(4):718-726. doi:10.1002/lio2.437 PMC7444791 32864444

[5] Khoury T Lavergne P Chitguppi C , et al. Aerosolized particle reduction: a novel cadaveric model and a negative airway pressure respirator (NAPR) system to protect health care workers from COVID-19. Otolaryngol Head Neck Surg. 2020;163(1):151-155. doi:10.1177/0194599820929275 32423338 PMC7240316

[6] Narwani V Kohli N Lerner MZ. Application of a modified endoscopy face mask for flexible laryngoscopy during the COVID-19 pandemic. Otolaryngol Head Neck Surg. 2020;163(1):107-109. doi:10.1177/0194599820928977 32423299

[7] Ference E Kim W Oghalai J Kim J Applegate B. COVID-19 in the clinic: aersol containment mask for endoscopic otolaryngologic procedures. Otolaryngol Head Neck Surg. doi:10.1177/01945998211024944 10.1177/01945998211024944 PMC8262032 34154484

[8] Kempster GB Gerratt BR Verdolini Abbott K Barkmeier-Kraemer J Hillman RE. Consensus auditory-perceptual evaluation of voice: development of a standardized clinical protocol. Am J Speech Language Pathol. 2009;18(2):124-132. doi:10.1044/1058-0360(2008/08-0017) 18930908

[9] de Araújo LMP Abatti PJ de Araújo Filho WD Alves RF. Performance evaluation of nebulizers based on aerodynamic droplet diameter characterization using the direct laminar incidence (DLI). Res Biomed Eng. 2017;33(2):105-112. doi:10.1590/2446-4740.05316

