# Supplementary material for: COVID-19 in the Clinic: Human Testing of an Aerosol Containment Mask for Endoscopic Clinic Procedures
Source: Otolaryngol Head Neck Surg. 2021 Jul 27;166(4):669–75. doi: 10.1177/01945998211029184 (PMC8978456; doi:10.1177/01945998211029184)
Supplement: sj-docx-1-oto-10.117701945998211029184 – Supplemental material for COVID-19 in the Clinic: Human Testing of an Aerosol Containment Mask for Endoscopic Clinic Procedures [file sj-docx-1-oto-10.117701945998211029184.docx]

| Volunteer | Location | Task | Mean | SD | SEM | P-value | Label |
| --- | --- | --- | --- | --- | --- | --- | --- |
| 1 | Sensor 1 | Normal (probe) | 46.91667 | 15.75694 | 2.03421 |  |  |
|  |  | Rainbow (probe) | 99.33333 | 20.36004 | 2.62847 | 0.0000 | ** |
|  |  | Normal (probe) | 53.15 | 23.66204 | 3.05476 |  |  |
|  |  | Cough (probe) | 120.75 | 27.48104 | 3.54779 | 0.0000 | ** |
|  |  | Normal (probe) | 48.55 | 14.89332 | 1.92272 |  |  |
|  |  | Sneeze (probe) | 185.3333 | 89.85443 | 11.60016 | 0.0000 | ** |
|  | Sensor 2 | Normal (probe) | 105.55 | 37.27108 | 4.81168 |  |  |
|  |  | Rainbow (probe) | 105.1667 | 25.13972 | 3.24552 | 0.9431 | * |
|  |  | Normal (probe) | 100.25 | 37.30617 | 4.81621 |  |  |
|  |  | Cough (probe) | 104.8667 | 40.93006 | 5.28405 | 0.4854 | * |
|  |  | Normal (probe) | 113.1833 | 31.3793 | 4.05105 |  |  |
|  |  | Sneeze (probe) | 94.15 | 40.4743 | 5.2251 | 0.0110 | ** |
|  | Sensor 1 | Normal (probe & suction) | 58.61667 | 22.63497 | 2.92216 |  |  |
|  |  | Rainbow (probe & suction) | 48.56667 | 25.59685 | 3.30454 | 0.0267 | ** |
|  |  | Normal (probe & suction) | 39.71667 | 17.26571 | 2.22899 |  |  |
|  |  | Cough (probe & scution) | 129.3667 | 51.19586 | 6.60936 | 0.0000 | ** |
|  |  | Normal (probe & suction) | 29.13333 | 17.29844 | 2.23322 |  |  |
|  |  | Sneeze (probe & suction) | 122.5 | 61.04055 | 7.8803 | 0.0000 | ** |
|  | Sensor 2 | Normal (probe & suction) | 157.5167 | 12.38437 | 1.59882 |  |  |
|  |  | Rainbow (probe & suction) | 161.6333 | 16.62953 | 2.14686 | 0.1565 | * |
|  |  | Normal (probe & suction) | 162.8667 | 13.55524 | 1.74997 |  |  |
|  |  | Cough (probe & suction) | 143.4 | 32.53327 | 4.20003 | 0.0000 | ** |
|  |  | Normal (probe & suction) | 172.4833 | 15.9931 | 2.0647 |  |  |
|  |  | Sneeze (probe & suction) | 157.4833 | 27.78275 | 3.58674 | 0.0008 | ** |
|  |  |  |  |  |  |  |  |
| 2 | Sensor 1 | Normal (probe) | 113.7333 | 33.42326 | 4.31492 |  |  |
|  |  | Rainbow (probe) | 174.6 | 48.6085 | 6.27533 | 0.0000 | ** |
|  |  | Normal (probe) | 88.93333 | 29.36322 | 3.79078 |  |  |
|  |  | Cough (probe) | 158.0667 | 80.64336 | 10.41101 | 0.0000 | ** |
|  |  | Normal (probe) | 101.3833 | 24.53506 | 3.16746 |  |  |
|  |  | Sneeze (probe) | 199.15 | 293.727 | 37.91999 | 0.0150 | ** |
|  | Sensor 2 | Normal (probe) | 126.6833 | 33.83734 | 4.36838 |  |  |
|  |  | Rainbow (probe) | 142.1 | 22.59571 | 2.91709 | 0.0045 | ** |
|  |  | Normal (probe) | 128.1333 | 34.66277 | 4.47494 |  |  |
|  |  | Cough (probe) | 110.1167 | 33.45125 | 4.31854 | 0.0027 | ** |
|  |  | Normal (probe) | 140.15 | 22.15571 | 2.86029 |  |  |
|  |  | Sneeze (probe) | 134.1333 | 33.59049 | 4.33651 | 0.2588 | * |
|  | Sensor 1 | Normal (probe & suction) | 56.58333 | 15.98547 | 2.06372 |  |  |
|  |  | Rainbow (probe & suction) | 50.41667 | 9.02969 | 1.16573 | 0.0138 | ** |
|  |  | Normal (probe & suction) | 33.16667 | 9.42619 | 1.21692 |  |  |
|  |  | Cough (probe & suction) | 197.75 | 280.6043 | 36.22586 | 0.0000 | ** |
|  |  | Normal (probe & suction) | 34.66667 | 10.83414 | 1.39868 |  |  |
|  |  | Sneeze (probe & suction) | 76.25 | 44.57887 | 5.75511 | 0.0000 | ** |
|  | Sensor 2 | Normal (probe & suction) | 161.9333 | 11.9602 | 1.54405 |  |  |
|  |  | Rainbow (probe & suction) | 158.9833 | 13.99091 | 1.80622 | 0.2290 | * |
|  |  | Normal (probe & suction) | 164 | 12.30406 | 1.58845 |  |  |
|  |  | Cough (probe & suction) | 131.6667 | 29.94552 | 3.86595 | 0.0000 | ** |
|  |  | Normal (probe & suction) | 163.7 | 14.68575 | 1.89592 |  |  |
|  |  | Sneeze (probe & suction) | 134.1333 | 33.59049 | 4.33651 | 0.0000 | ** |
|  |  |  |  |  |  |  |  |
| 3 | Sensor 1 | Normal (probe) | 60.18333 | 16.8538 | 2.17582 |  |  |
|  |  | Rainbow (probe) | 159.1667 | 37.10643 | 4.79042 | 0.0000 | ** |
|  |  | Normal (probe) | 62.28333 | 10.74684 | 1.38741 |  |  |
|  |  | Cough (probe) | 267.1167 | 154.1318 | 19.89833 | 0.0000 | ** |
|  |  | Normal (probe) | 60.96667 | 17.02637 | 2.19809 |  |  |
|  |  | Sneeze (probe) | 1210.75 | 2152.11 | 277.8362 | 0.0001 | ** |
|  | Sensor 2 | Normal (probe) | 163.8833 | 27.47208 | 3.54663 |  |  |
|  |  | Rainbow (probe) | 165.45 | 22.15923 | 2.86074 | 0.7350 | * |
|  |  | Normal (probe) | 146.65 | 23.21004 | 2.9964 |  |  |
|  |  | Cough (probe) | 137.9 | 32.01679 | 4.13335 | 0.1237 | * |
|  |  | Normal (probe) | 141.65 | 25.86066 | 3.3386 |  |  |
|  |  | Sneeze (probe) | 148.2667 | 27.7671 | 3.5847 | 0.1817 | * |
|  | Sensor 1 | Normal (probe & suction) | 16.2 | 7.24581 | 0.93543 |  |  |
|  |  | Rainbow (probe & suction) | 22.73333 | 9.90098 | 1.27821 | 0.0001 | ** |
|  |  | Normal (probe & suction) | 11.51667 | 11.46918 | 1.48066 |  |  |
|  |  | Cough (probe & suction) | 202.6833 | 180.3695 | 23.28561 | 0.0000 | ** |
|  |  | Normal (probe & suction) | 15.43333 | 14.42851 | 1.86271 |  |  |
|  |  | Sneeze (probe & suction) | 355.55 | 693.045 | 89.47172 | 0.0003 | ** |
|  | Sensor 2 | Normal (probe & suction) | 163.2833 | 11.54078 | 1.48991 |  |  |
|  |  | Rainbow (probe & suction) | 133.75 | 31.41554 | 4.05573 | 0.0000 | ** |
|  |  | Normal (probe & suction) | 147.0833 | 23.94718 | 3.09157 |  |  |
|  |  | Cough (probe & suction) | 138.5667 | 25.43011 | 3.28301 | 0.0941 | * |
|  |  | Normal (probe & suction) | 165.2833 | 13.20515 | 1.70478 |  |  |
|  |  | Sneeze (probe & suction) | 164.8667 | 11.89141 | 1.53517 | 0.8568 | * |
|  |  |  |  |  |  |  |  |
| 4 | Sensor 1 | Normal (probe) | 54.91667 | 10.23833 | 1.32176 |  |  |
|  |  | Rainbow (probe) | 102.2333 | 14.38734 | 1.8574 | 0.0000 | ** |
|  |  | Normal (probe) | 45.93333 | 9.7596 | 1.25996 |  |  |
|  |  | Cough (probe) | 94.66667 | 29.08297 | 3.7546 | 0.0000 | ** |
|  |  | Normal (probe) | 41.4 | 8.00466 | 1.0334 |  |  |
|  |  | Sneeze (probe) | 111.0833 | 50.41667 | 6.50876 | 0.0000 | ** |
|  | Sensor 2 | Normal (probe) | 148.4 | 52.17903 | 6.73628 |  |  |
|  |  | Rainbow (probe) | 157.05 | 30.29763 | 3.91141 | 0.3020 | * |
|  |  | Normal (probe) | 163.3833 | 29.05873 | 3.75147 |  |  |
|  |  | Cough (probe) | 156.8833 | 35.81122 | 4.62321 | 0.3363 | * |
|  |  | Normal (probe) | 144.4333 | 31.78443 | 4.10335 |  |  |
|  |  | Sneeze (probe) | 117.55 | 23.44949 | 3.02732 | 0.0000 | ** |
|  | Sensor 1 | Normal (probe & suction) | 39.06667 | 8.72143 | 1.12593 |  |  |
|  |  | Rainbow (probe & suction) | 31.13333 | 6.34453 | 0.81908 | 0.0000 | ** |
|  |  | Normal (probe & suction) | 26.9 | 6.15258 | 0.79429 |  |  |
|  |  | Cough (probe & suction) | 38.48333 | 16.1775 | 2.08851 | 0.0000 | ** |
|  |  | Normal (probe & suction) | 22.06667 | 4.7474 | 0.61289 |  |  |
|  |  | Sneeze (probe & suction) | 114.1 | 182.546 | 23.56659 | 0.0002 | ** |
|  | Sensor 2 | Normal (probe & suction) | 150.5333 | 19.17225 | 2.47513 |  |  |
|  |  | Rainbow (probe & suction) | 151.3333 | 12.05168 | 1.55586 | 0.7809 | * |
|  |  | Normal (probe & suction) | 147.0167 | 11.98232 | 1.54691 |  |  |
|  |  | Cough (probe & suction) | 123.7333 | 24.8677 | 3.21041 | 0.0000 | ** |
|  |  | Normal (probe & suction) | 138.7833 | 13.72254 | 1.77157 |  |  |
|  |  | Sneeze (probe & suction) | 117.55 | 23.44949 | 3.02732 | 0.0000 | ** |
|  |  |  |  |  |  |  |  |
| 5 | Sensor 1 | Normal (probe) | 84.5 | 17.72913 | 2.28882 |  |  |
|  |  | Rainbow (probe) | 157.2833 | 32.0371 | 4.13597 | 0.0000 | ** |
|  |  | Normal (probe) | 103.8833 | 21.92057 | 2.82993 |  |  |
|  |  | Cough (probe) | 1235.9 | 1377.094 | 177.7821 | 0.0000 | ** |
|  |  | Normal (probe) | 128.6333 | 32.02434 | 4.13432 |  |  |
|  |  | Sneeze (probe) | 346.8333 | 422.7976 | 54.58293 | 0.0002 | ** |
|  | Sensor 2 | Normal (probe) | 172.2333 | 48.4304 | 6.25234 |  |  |
|  |  | Rainbow (probe) | 168.2167 | 27.97401 | 3.61143 | 0.5649 | * |
|  |  | Normal (probe) | 180.2333 | 38.49956 | 4.97027 |  |  |
|  |  | Cough (probe) | 182.8333 | 38.14476 | 4.92447 | 0.7555 | * |
|  |  | Normal (probe) | 154.9333 | 65.76972 | 8.49083 |  |  |
|  |  | Sneeze (probe) | 178.0333 | 37.12323 | 4.79259 | 0.0260 | ** |
|  | Sensor 1 | Normal (probe & suction) | 70.83333 | 15.94287 | 2.05822 |  |  |
|  |  | Rainbow (probe & suction) | 76.78333 | 16.8232 | 2.17187 | 0.0445 | ** |
|  |  | Normal (probe & suction) | 58.05 | 15.71292 | 2.02853 |  |  |
|  |  | Cough (probe & suction) | 728.5833 | 896.2798 | 115.7092 | 0.0000 | ** |
|  |  | Normal (probe & suction) | 73.4 | 20.27957 | 2.61808 |  |  |
|  |  | Sneeze (probe & suction) | 264.5333 | 107.2353 | 13.84402 | 0.0000 | ** |
|  | Sensor 2 | Normal (probe & suction) | 216.35 | 17.97345 | 2.32036 |  |  |
|  |  | Rainbow (probe & suction) | 215.7167 | 13.63332 | 1.76005 | 0.8152 | * |
|  |  | Normal (probe & suction) | 212.35 | 15.39186 | 1.98708 |  |  |
|  |  | Cough (probe & suction) | 179.8167 | 37.67501 | 4.86382 | 0.0000 | ** |
|  |  | Normal (probe & suction) | 210.6 | 13.43433 | 1.73437 |  |  |
|  |  | Sneeze (probe & suction) | 178.0333 | 37.12323 | 4.79259 | 0.0000 | ** |
|  |  |  |  |  |  |  |  |
| 6 | Sensor 1 | Normal (probe) | 184.5 | 56.22518 | 7.25864 |  |  |
|  |  | Rainbow (probe) | 310.1167 | 71.44444 | 9.22344 | 0.0000 | ** |
|  |  | Normal (probe) | 235.0333 | 71.16035 | 9.18676 |  |  |
|  |  | Cough (probe) | 508.3667 | 286.5322 | 36.99114 | 0.0000 | ** |
|  |  | Normal (probe) | 246.7 | 69.79517 | 9.01052 |  |  |
|  |  | Sneeze (probe) | 399.0667 | 130.3319 | 16.82577 | 0.0000 | ** |
|  | Sensor 2 | Normal (probe) | 193.9667 | 35.19002 | 4.54301 |  |  |
|  |  | Rainbow (probe) | 194.85 | 31.99514 | 4.13055 | 0.8644 | * |
|  |  | Normal (probe) | 183.7333 | 43.17323 | 5.57364 |  |  |
|  |  | Cough (probe) | 197.4667 | 42.71882 | 5.51498 | 0.0672 | * |
|  |  | Normal (probe) | 212.15 | 31.98878 | 4.12973 |  |  |
|  |  | Sneeze (probe) | 203.2833 | 59.6192 | 7.6968 | 0.3071 | * |
|  | Sensor 1 | Normal (probe & suction) | 161.1167 | 33.60089 | 4.33786 |  |  |
|  |  | Rainbow (probe & suction) | 141.7 | 36.8245 | 4.75402 | 0.0047 | ** |
|  |  | Normal (probe & suction) | 98.11667 | 41.13973 | 5.31112 |  |  |
|  |  | Cough (probe & suction) | 284.9833 | 120.6787 | 15.57955 | 0.0000 | ** |
|  |  | Normal (probe & suction) | 77.1 | 32.18416 | 4.15496 |  |  |
|  |  | Sneeze (probe & suction) | 378.0833 | 155.165 | 20.03172 | 0.0000 | ** |
|  | Sensor 2 | Normal (probe & suction) | 405.4833 | 21.23915 | 2.74196 |  |  |
|  |  | Rainbow (probe & suction) | 430.5833 | 28.39569 | 3.66587 | 0.0000 | ** |
|  |  | Normal (probe & suction) | 435.7167 | 31.14759 | 4.02114 |  |  |
|  |  | Cough (probe & suction) | 432.2333 | 50.38676 | 6.5049 | 0.6730 | * |
|  |  | Normal (probe & suction) | 471.2667 | 27.31292 | 3.52608 |  |  |
|  |  | Sneeze (probe & suction) | 443.8833 | 65.0713 | 8.40067 | 0.0050 | ** |
|  |  |  |  |  |  |  |  |
| 7 | Sensor 1 | Normal (probe) | 454.1833 | 112.3215 | 14.50064 |  |  |
|  |  | Rainbow (probe) | 537.5833 | 161.3654 | 20.83218 | 0.0002 | ** |
|  |  | Normal (probe) | 434.1 | 96.0259 | 12.39689 |  |  |
|  |  | Cough (probe) | 673.2333 | 201.5668 | 26.02216 | 0.0000 | ** |
|  |  | Normal (probe) | 306.5167 | 82.00568 | 10.58689 |  |  |
|  |  | Sneeze (probe) | 630.75 | 212.4253 | 27.42398 | 0.0000 | ** |
|  | Sensor 2 | Normal (probe) | 647.6333 | 78.46471 | 10.12975 |  |  |
|  |  | Rainbow (probe) | 656.5167 | 66.04454 | 8.52631 | 0.5143 | * |
|  |  | Normal (probe) | 683.75 | 73.09609 | 9.43666 |  |  |
|  |  | Cough (probe) | 652.1 | 105.8755 | 13.66847 | 0.0464 | * |
|  |  | Normal (probe) | 674.9167 | 115.1646 | 14.86769 |  |  |
|  |  | Sneeze (probe) | 680.85 | 179.0573 | 23.1162 | 0.8290 | * |
|  | Sensor 1 | Normal (probe & suction) | 289.9333 | 60.79721 | 7.84889 |  |  |
|  |  | Rainbow (probe & suction) | 245.75 | 37.24615 | 4.80846 | 0.0000 | ** |
|  |  | Normal (probe & suction) | 212.0333 | 81.06076 | 10.4649 |  |  |
|  |  | Cough (probe & suction) | 398.7667 | 101.6152 | 13.11846 | 0.0000 | ** |
|  |  | Normal (probe & suction) | 179.9667 | 58.97025 | 7.61303 |  |  |
|  |  | Sneeze (probe & suction) | 155.65 | 44.68945 | 5.76938 | 0.0092 | ** |
|  | Sensor 2 | Normal (probe & suction) | 818.6167 | 37.6145 | 4.85601 |  |  |
|  |  | Rainbow (probe & suction) | 829.2167 | 34.14629 | 4.40827 | 0.1667 | * |
|  |  | Normal (probe & suction) | 830.3167 | 32.33851 | 4.17488 |  |  |
|  |  | Cough (probe & suction) | 771.3333 | 97.5779 | 12.59725 | 0.0000 | ** |
|  |  | Normal (probe & suction) | 783.6333 | 84.72707 | 10.93822 |  |  |
|  |  | Sneeze (probe & suction) | 680.85 | 179.0573 | 23.1162 | 0.0003 | ** |
|  |  |  |  |  |  |  |  |
| 8 | Sensor 1 | Normal (probe) | 747.25 | 146.8545 | 73.42726 |  |  |
|  |  | Rainbow (probe) | 1340.75 | 187.0105 | 93.50524 | 0.0007 | ** |
|  |  | Normal (probe) | 689.25 | 54.35915 | 27.17957 |  |  |
|  |  | Cough (probe) | 1458.25 | 657.9804 | 328.9902 | 0.1025 | * |
|  |  | Normal (probe) | 475.75 | 78.47877 | 39.23938 |  |  |
|  |  | Sneeze (probe) | 1072.75 | 242.2318 | 121.1159 | 0.0286 | ** |
|  | Sensor 2 | Normal (probe) | 207.75 | 38.84547 | 5.01493 |  |  |
|  |  | Rainbow (probe) | 235.45 | 18.81527 | 2.42904 | 0.0000 | ** |
|  |  | Normal (probe) | 219.1333 | 26.99998 | 3.48568 |  |  |
|  |  | Cough (probe) | 223.7333 | 27.03787 | 3.49057 | 0.3751 | * |
|  |  | Normal (probe) | 185.0167 | 28.55295 | 3.68617 |  |  |
|  |  | Sneeze (probe) | 232.45 | 23.29067 | 3.00681 | 0.0000 | ** |
|  | Sensor 1 | Normal (probe & suction) | 124 | 49.38961 | 24.6948 |  |  |
|  |  | Rainbow (probe & suction) | 122.25 | 20.3695 | 10.18475 | 0.9436 | * |
|  |  | Normal (probe & suction) | 71.5 | 10.47219 | 5.23609 |  |  |
|  |  | Cough (probe & suction) | 267.25 | 31.25567 | 15.62783 | 0.0014 | ** |
|  |  | Normal (probe & suction) | 101.25 | 35.54692 | 17.77346 |  |  |
|  |  | Sneeze (probe & suction) | 519.25 | 367.6043 | 183.8022 | 0.1245 | * |
|  | Sensor 2 | Normal (probe & suction) | 242.7 | 15.41989 | 1.9907 |  |  |
|  |  | Rainbow (probe & suction) | 239.0833 | 12.73763 | 1.64442 | 0.1696 | * |
|  |  | Normal (probe & suction) | 240.1833 | 19.10319 | 2.46621 |  |  |
|  |  | Cough (probe & suction) | 227.85 | 34.64093 | 4.47212 | 0.0212 | ** |
|  |  | Normal (probe & suction) | 237.0667 | 16.64675 | 2.14909 |  |  |
|  |  | Sneeze (probe & suction) | 232.45 | 23.29067 | 3.00681 | 0.2205 | * |
|  |  |  |  |  |  |  |  |
| 9 | Sensor 1 | Normal (probe) | 888 | 80.21222 | 40.10611 |  |  |
|  |  | Rainbow (probe) | 2887.25 | 393.4271 | 196.7136 | 0.0031 | ** |
|  |  | Normal (probe) | 601.5 | 71.45395 | 35.72697 |  |  |
|  |  | Cough (probe) | 823.25 | 77.60316 | 38.80158 | 0.0365 | ** |
|  |  | Normal (probe) | 473.75 | 50.1157 | 25.05785 |  |  |
|  |  | Sneeze (probe) | 1280.5 | 434.4264 | 217.2132 | 0.0305 | ** |
|  | Sensor 2 | Normal (probe) | 206.2667 | 19.65798 | 2.53783 |  |  |
|  |  | Rainbow (probe) | 212.3167 | 21.11269 | 2.72564 | 0.1060 | * |
|  |  | Normal (probe) | 209.5667 | 21.66335 | 2.79673 |  |  |
|  |  | Cough (probe) | 216.0667 | 19.57763 | 2.52746 | 0.0944 | * |
|  |  | Normal (probe) | 181.0333 | 31.29952 | 4.04075 |  |  |
|  |  | Sneeze (probe) | 216.05 | 15.25981 | 1.97003 | 0.0000 | ** |
|  | Sensor 1 | Normal (probe & suction) | 569 | 50.9575 | 25.47875 |  |  |
|  |  | Rainbow (probe & suction) | 1018.25 | 102.3926 | 51.19631 | 0.0089 | ** |
|  |  | Normal (probe & suction) | 556.75 | 30.60909 | 15.30455 |  |  |
|  |  | Cough (probe & suction) | 1245 | 317.7473 | 158.8736 | 0.0184 | ** |
|  |  | Normal (probe & suction) | 723.25 | 124.4706 | 62.23527 |  |  |
|  |  | Sneeze (probe & suction) | 1340.75 | 187.0105 | 93.50524 | 0.0124 | ** |
|  | Sensor 2 | Normal (probe & suction) | 206.6667 | 27.84075 | 3.59423 |  |  |
|  |  | Rainbow (probe & suction) | 205.4 | 29.81821 | 3.84951 | 0.8059 | * |
|  |  | Normal (probe & suction) | 194.1 | 27.32274 | 3.52735 |  |  |
|  |  | Cough (probe & suction) | 220.15 | 14.95624 | 1.93084 | 0.0000 | ** |
|  |  | Normal (probe & suction) | 216.1 | 16.82683 | 2.17234 |  |  |
|  |  | Sneeze (probe & suction) | 216.05 | 15.25981 | 1.97003 | 0.9868 | * |
|  |  |  |  |  |  |  |  |
| 10 | Sensor 1 | Normal (probe) | 28.75 | 8.45251 | 1.09121 |  |  |
|  |  | Rainbow (probe) | 75.5 | 26.65505 | 3.44115 | 0.0000 | ** |
|  |  | Normal (probe) | 46.46667 | 14.72973 | 1.9016 |  |  |
|  |  | Cough (probe) | 129.5667 | 76.43018 | 9.86709 | 0.0000 | ** |
|  |  | Normal (probe) | 35.6 | 10.81462 | 1.39616 |  |  |
|  |  | Sneeze (probe) | 582.4833 | 953.6997 | 123.1221 | 0.0000 | ** |
|  | Sensor 2 | Normal (probe) | 103.3 | 14.42843 | 1.8627 |  |  |
|  |  | Rainbow (probe) | 98.25 | 16.13988 | 2.08365 | 0.0658 | * |
|  |  | Normal (probe) | 105 | 15.79637 | 2.0393 |  |  |
|  |  | Cough (probe) | 105.85 | 17.84949 | 2.30436 | 0.7694 | * |
|  |  | Normal (probe) | 92.25 | 22.96764 | 2.96511 |  |  |
|  |  | Sneeze (probe) | 114.35 | 21.48932 | 2.77426 | 0.0000 | ** |
|  | Sensor 1 | Normal (probe & suction) | 16.28333 | 6.06767 | 0.78333 |  |  |
|  |  | Rainbow (probe & suction) | 22.01667 | 6.49509 | 0.83851 | 0.0000 | ** |
|  |  | Normal (probe & suction) | 17.08333 | 9.73425 | 1.25669 |  |  |
|  |  | Cough (probe & suction) | 85.96667 | 95.72063 | 12.35748 | 0.0000 | ** |
|  |  | Normal (probe & suction) | 12.98333 | 3.78441 | 0.48857 |  |  |
|  |  | Sneeze (probe & suction) | 128 | 137.5617 | 17.75914 | 0.0000 | ** |
|  | Sensor 2 | Normal (probe & suction) | 121.1667 | 10.77846 | 1.39149 |  |  |
|  |  | Rainbow (probe & suction) | 123.45 | 10.28645 | 1.32797 | 0.2502 | * |
|  |  | Normal (probe & suction) | 118.6 | 12.16998 | 1.57114 |  |  |
|  |  | Cough (probe & suction) | 118.7 | 17.77572 | 2.29484 | 0.9711 | * |
|  |  | Normal (probe & suction) | 131.95 | 11.54714 | 1.49073 |  |  |
|  |  | Sneeze (probe & suction) | 114.35 | 21.48932 | 2.77426 | 0.0000 | ** |
