# Supplementary figures and images for: COVID-19 in the Clinic: Human Testing of an Aerosol Containment Mask for Endoscopic Clinic Procedures
Source: Otolaryngol Head Neck Surg. 2021 Jul 27;166(4):669–75. doi: 10.1177/01945998211029184 (PMC8978456; doi:10.1177/01945998211029184)

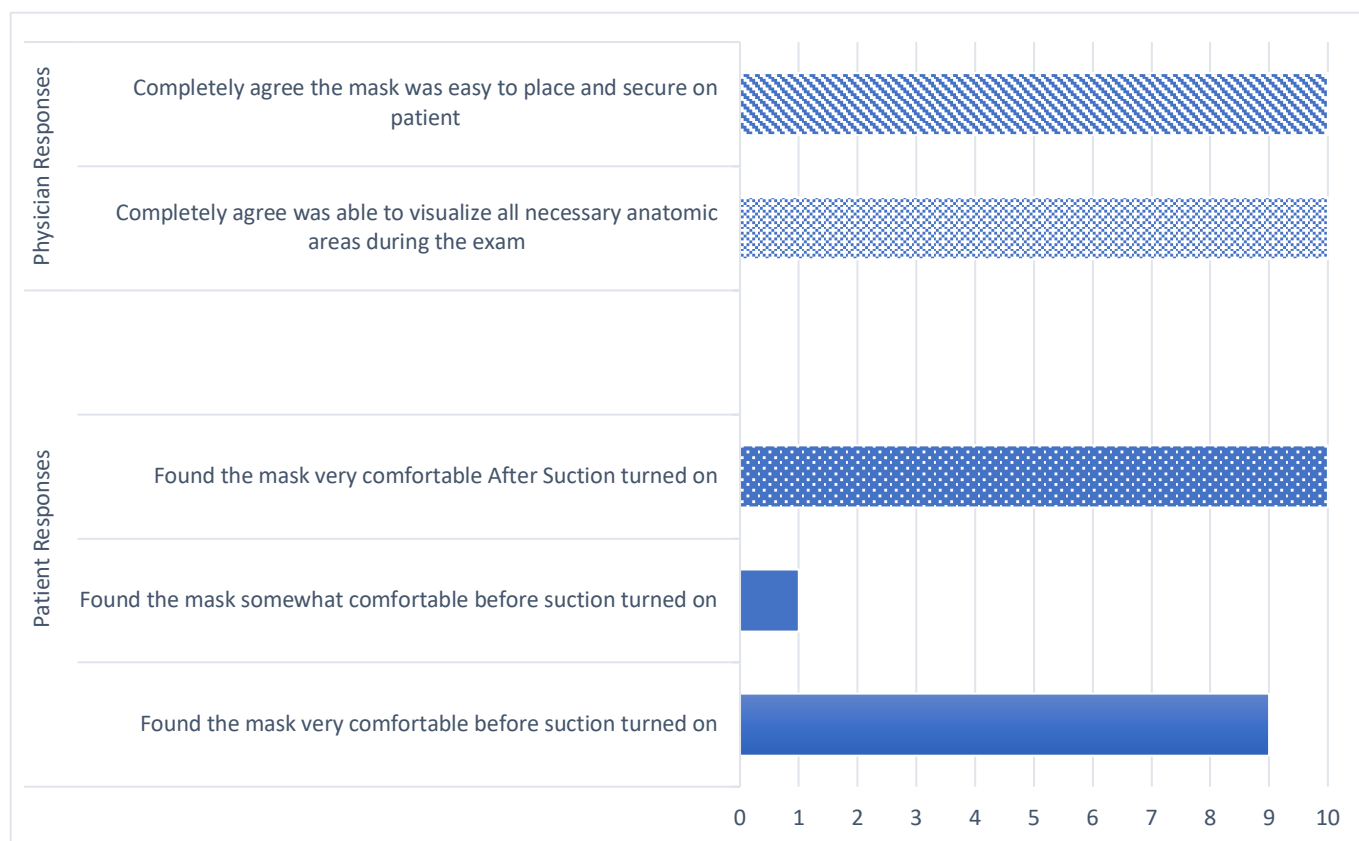

Supplement: sj-pdf-2-oto-10.117701945998211029184 – Supplemental material for COVID-19 in the Clinic: Human Testing of an Aerosol Containment Mask for Endoscopic Clinic Procedures [file sj-pdf-2-oto-10.117701945998211029184.pdf]
